# Prospects of Using Chitosan-Based Biopolymers in the Treatment of Peripheral Nerve Injuries

**DOI:** 10.3390/ijms241612956

**Published:** 2023-08-19

**Authors:** Meng Zhang, Heng An, Fengshi Zhang, Haoran Jiang, Teng Wan, Yongqiang Wen, Na Han, Peixun Zhang

**Affiliations:** 1 Department of Orthopedics and Trauma, Peking University People’s Hospital, Beijing 100044, China; mengzh2008@bjmu.edu.cn (M.Z.);; 2 Key Laboratory of Trauma and Neural Regeneration, Ministry of Education, Beijing 100044, China; 3 Beijing Key Laboratory for Bioengineering and Sensing Technology, Daxing Research Institute, School of Chemistry & Biological Engineering, University of Science & Technology Beijing, Beijing 100083, China; hengan@xs.ustb.edu.cn (H.A.);

**Keywords:** chitosan, chitosan-based biopolymers, functionalized polymers, carbohydrate polymers, peripheral nerve injury

## Abstract

Peripheral nerve injuries are common neurological disorders, and the available treatment options, such as conservative management and surgical repair, often yield limited results. However, there is growing interest in the potential of using chitosan-based biopolymers as a novel therapeutic approach to treating these injuries. Chitosan-based biopolymers possess unique characteristics, including biocompatibility, biodegradability, and the ability to stimulate cell proliferation, making them highly suitable for repairing nerve defects and promoting nerve regeneration and functional recovery. Furthermore, these biopolymers can be utilized in drug delivery systems to control the release of therapeutic agents and facilitate the growth of nerve cells. This comprehensive review focuses on the latest advancements in utilizing chitosan-based biopolymers for peripheral nerve regeneration. By harnessing the potential of chitosan-based biopolymers, we can pave the way for innovative treatment strategies that significantly improve the outcomes of peripheral nerve injury repair, offering renewed hope and better prospects for patients in need.

## 1. Introduction

Peripheral nerve injuries (PNIs) pose significant challenges to patients and healthcare professionals alike. They can result in pain, loss of motor function, and sensory deficits, impacting an individual’s quality of life [1,2,3]. However, recent advancements in the understanding and treatment of PNIs have opened up new possibilities for improved outcomes. Surgical intervention remains the primary treatment option for severe PNIs [4]. Nerve repair techniques have evolved significantly, with microsurgical approaches becoming the gold standard. Direct end-to-end neurorrhaphy, nerve grafting, nerve transfers, and tubulization procedures are commonly employed. The development of bioengineered nerve conduits and scaffolds has shown promise in bridging larger nerve gaps, reducing donor site morbidity, and promoting nerve regeneration [5,6,7]. Recent advances in the field of neural regeneration have led to promising therapeutic approaches. For example, through tissue engineering approaches, tissue-engineered nerve grafts composed of natural or synthetic materials combined with stem cells and growth factors have shown potential in promoting nerve regeneration [8,9,10].

**Figure 1:**
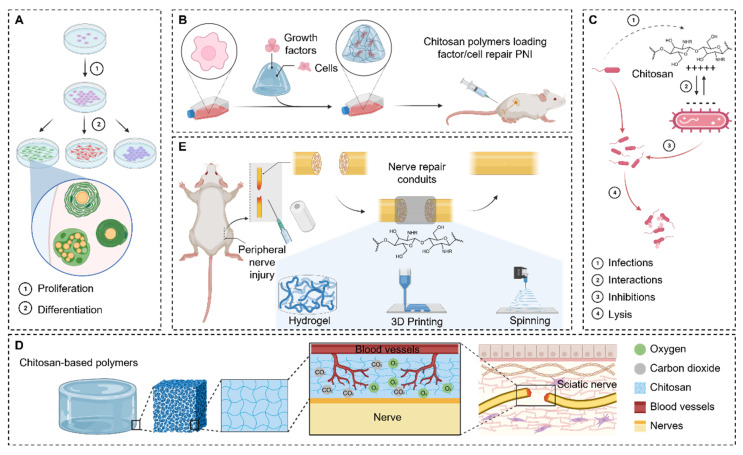
The chitosan-based biopolymer repair of a peripheral nerve injury. (**A**) Chitosan-based polymers have excellent biocompatibility. (**B**) Chitosan-based polymer materials can act as carriers of loaded cells and factors to promote peripheral nerve regeneration. (**C**) Chitosan has excellent antibacterial properties. (**D**) The porous structure of chitosan-based polymers facilitates vascular regeneration and the exchange of oxygen and nutrients. (**E**) Patterns of peripheral nerve injury treated with chitosan based biopolymers.

Chitosan plays a crucial role as an important component of tissue engineering materials for peripheral nerve repair [11]. As a biodegradable polymer, chitosan possesses several remarkable characteristics that make it an ideal choice in the field of nerve regeneration (Figure 1A) [12]. First, chitosan exhibits excellent biocompatibility, allowing it to interact with surrounding tissues and support cell growth and differentiation (Figure 1B). Second, it can form a three-dimensional scaffold structure that provides support and guidance for nerve cells, promoting nerve regeneration with a pronounced antibacterial effect (Figure 1C) [13,14]. Moreover, chitosan possesses a porous structure that facilitates the transport of nutrients and oxygen, enhancing the growth of nerve cells and the formation of new blood vessels (Figure 1D) [15,16]. Additionally, chitosan can interact with growth factors and cytokines, boosting the proliferation and differentiation of nerve cells and regulating the process of nerve regeneration [17,18,19,20]. Therefore, chitosan serves as a vital component in tissue engineering materials used for peripheral nerve repair, providing an ideal tool and environment for nerve regeneration (Figure 1E).

## 2. Treatment of Peripheral Nerve Injuries

### 2.1. Neural Epithelial Suture

**Figure 2:**
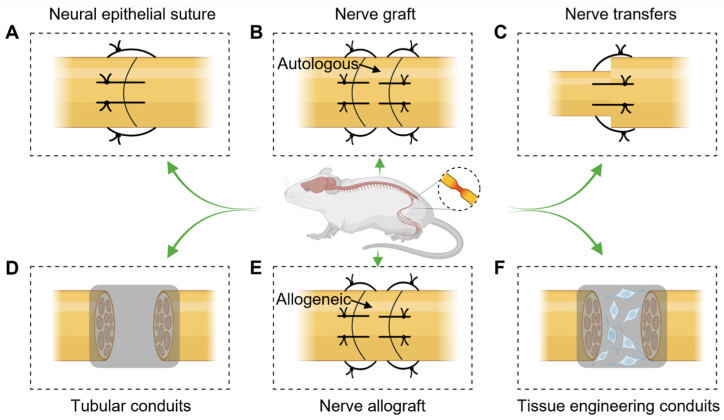
Different ways to repair peripheral nerve injuries. (**A**) Neural epithelial suture. (**B**) Nerve graft. (**C**) Nerve transfers. (**D**) Tubular conduits. (**E**) Nerve allograft. (**F**) Tissue engineering conduits.

Neurorrhaphy, also known as nerve epineurial suturing, is a commonly used technique for repairing peripheral nerve injuries. By precisely suturing the damaged nerve epineurium, it aims to restore the continuity and function of the nerve, promoting nerve regeneration and recovery (Figure 2A). This method provides support and protection to assist the damaged nerve in regaining normal conduction capabilities. Peripheral nerve injuries can result from external trauma, surgical procedures, or diseases, or events such as lacerations, crush injuries, or traction injuries. During the surgical procedure, the surgeon must demonstrate a high level of skill and precision to align and suture the damaged nerve endings. Following the repair, patients typically undergo rehabilitation and recovery training, including physical therapy and rehabilitation exercises, to aid in the restoration of nerve function. Neurorrhaphy offers patients an opportunity to improve their quality of life and regain normal nerve functionality. However, it is important to note that neurorrhaphy requires significant surgical expertise and is limited in cases involving long nerve gaps. Generally, nerve gaps larger than 2 mm cannot be repaired via direct tension-free suturing, which significantly limits the application of neurorrhaphy.

### 2.2. Nerve Grafting

Nerve grafting is a commonly used method for repairing peripheral nerve injuries. This technique involves transplanting donor nerve tissue to the damaged nerve site to restore nerve continuity and function, promoting nerve regeneration and recovery. Donor nerves can be obtained from other parts of the same patient (autograft) or from another individual (allografts) (Figure 2B). During surgery, the surgeon precisely connects the donor nerve tissue to the damaged nerve endings or defects. Nerve grafting is suitable for larger nerve defects and provides a larger supply of nerve tissue. Additionally, the introduction of donor nerve tissue facilitates the delivery of nutrients and growth factors, aiding in nerve regeneration. However, this method involves challenges such as donor nerve selection, compatibility, and the need to overcome immune rejection. Furthermore, the donor site may experience disability and pain. Nerve grafting is a reparative technique used for peripheral nerve injuries, but further research and practice are needed to optimize its application.

### 2.3. Nerve Transfers

Nerve transfer repair is a less commonly used method for repairing peripheral nerve injuries. It is employed only when the direct end-to-end suturing of the same nerve is not possible. In some cases, such as intercostal muscle to myocutaneous nerve transfers, a thinner proximal nerve is connected to a thicker distal nerve, restoring nerve continuity and functionality (Figure 2C). This method relies on the brain’s neuroplasticity, allowing patients to achieve the reinnervation of the distal muscles through repeated practice. However, the use of a thinner donor nerve results in a lower proportion of axons being available for reinnervation compared to the original nerve, limiting the effective restoration of limb function. Additionally, the incapacitation and pain in the donor nerve’s innervation area also pose significant limitations to its application.

### 2.4. Tubular Conduits

Tubular conduits are an innovative biomedical engineering technology used to repair peripheral nerve injuries. Tubular conduits, made from materials with high biocompatibility, are tube-like structures precisely placed at the site of damaged nerves (Figure 2D). These conduits not only protect the injured nerves from further damage but also provide a scaffold to promote nerve regeneration and healing. Material selection takes into consideration the biocompatibility, biodegradability, and interactions with surrounding tissues to maximize the repair process. During the repair process, the interior of the conduit provides a guided pathway, encouraging nerve endings to reconnect and bridge the gap in the damaged area. This guiding feature is crucial for nerve regeneration, as natural nerve regrowth often lacks accuracy and directionality. Scientists and doctors continue to work on improving the design, materials, and application of bioactive substances in these conduits to enhance treatment success and benefit more patients from this innovative therapy.

### 2.5. Nerve Allograft

Nerve allograft is a biomedical technique used for repairing peripheral nerve injuries. It involves transferring allogeneic nerve tissue, which has been rendered non-immunogenic, to a damaged nerve in a recipient, with the aim of promoting nerve regeneration and restoring function (Figure 2E). This approach is particularly suitable for severe nerve injuries, especially when there is a long gap in the damaged nerve or when other nerve repair methods have been unsuccessful. Nerve allografts are typically sourced from organ donors, and the donor nerve tissue must undergo a series of procedures to reduce the risk of rejection and transplant failure. These grafts offer unique advantages in nerve repair. Compared to synthetic materials, they provide a structure and biological characteristics that closely resemble natural nerves, creating a more conducive environment for nerve regeneration. Additionally, nerve allografts can provide essential support and guidance, facilitating the proper growth of regenerating nerves and aiding in the restoration of normal nerve function. Overall, nerve allografts hold promising prospects as a method for repairing complex and severe peripheral nerve injuries.

### 2.6. Tissue Engineering Conduits

Tissue engineering nerve conduits are an advanced method for repairing peripheral nerve injuries. They utilize artificially constructed conduits as scaffolds to provide a favorable environment for nerve regeneration, promoting nerve continuity and functional recovery. The conduits are typically made of biocompatible materials, with an internal structure that features micropores and fiber networks, mimicking the characteristics of natural nerve tissue. This design facilitates cell attachment, proliferation, and the growth and guidance of nerve fibers. During surgery, the conduits are placed at the site of the damaged nerve defect, providing accurate alignment and support to promote the migration and growth of nerve cells, restoring normal conduction function (Figure 2F). Additionally, the conduits help to prevent the collapse and adhesion of the surrounding structures, providing a favorable environment for nerve regeneration. The application of tissue engineering nerve conduits has the potential to optimize the repair of nerve injuries and offers new hope for functional recovery in patients. Future research will further improve conduit design and material selection, enhancing the effectiveness of repairs and facilitating successful nerve regeneration. Among the relevant materials, chitosan-based biopolymers are important materials in tissue engineering nerve conduits. Many commercially available conduits that have been introduced to clinical treatments, as well as materials that are still in the experimental research phase, are based on chitosan.

## 3. Overview of Chitosan-Based Polymers and Their Properties

Chitosan-based biopolymers are advanced materials that are widely used to repair peripheral nerve injuries. They exhibit excellent biocompatibility, allowing for favorable interactions with surrounding tissues and cells without triggering immune reactions or rejections. This makes chitosan-based biopolymers an ideal choice for supporting and promoting nerve regeneration. Additionally, they possess antimicrobial properties, effectively inhibiting bacterial growth and adhesion, reducing the risk of infections, and safeguarding the integrity of damaged nerves and surrounding tissues. The porous structure of chitosan-based biopolymers is another advantage, providing an optimal environment for cell attachment and growth. These pores can be utilized to load cells, growth factors, or other bioactive substances to facilitate nerve regeneration and recovery. By controlling the size and distribution of the pores, the release rate of cells and factors can be modulated, allowing for targeted and sustained support for nerve regeneration. In conclusion, chitosan-based biopolymers exhibit outstanding biocompatibility, antimicrobial properties, and a porous structure, making them an ideal material for repairing peripheral nerve injuries. Ongoing research and development will further optimize the characteristics and performance of chitosan-based biopolymers, facilitating their application in the field of nerve injury repair.

### 3.1. Biocompatibility

**Figure 3:**
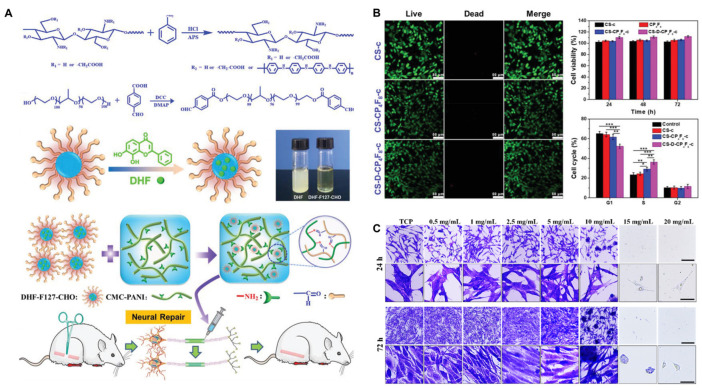
Chitosan-based polymers have excellent biocompatibility. (**A**) The synthetic route of a polyaniline-modified carboxymethyl chitosan (CMC-PANI). (**B**) LIVE/DEAD staining of RSC-96 cells treated with CS-c, CS-CP4F8-c, and CS-D-CP4F8-c. (* *p* < 0.05, ** *p* < 0.01, and *** *p* < 0.001) (**C**) Representative crystal violet staining images of PC12 cultured on TCP, 0.5 mg/mL, 1 mg/mL, 2.5 mg/mL, 5 mg/mL, 10 mg/mL, 15 mg/mL, and 20 mg/mL pDScNM for 24 h and 72 h. Copyright reprinted with permission from [22,23].

Chitosan exhibits excellent biocompatibility, allowing it to seamlessly interact with the surrounding tissues and effectively support cellular growth and differentiation. Its biocompatible nature ensures that it will produce minimal adverse reactions or immune responses when implanted into the body. The surface properties of chitosan enable favorable interactions with cells, facilitating cell adhesion and promoting cell proliferation [21,22]. Moreover, chitosan’s unique molecular structure provides binding sites for cell receptors, promoting cell signaling and triggering the specific cellular responses necessary for tissue regeneration [23]. This favorable interaction between chitosan and the surrounding tissue creates an optimal microenvironment in which cells can thrive, ensuring their viability and fostering their differentiation into specialized nerve cells [14,24,25,26,27]. As shown in Figure 3, the biocompatible nature of chitosan, combined with its ability to support cell growth and differentiation, makes it a highly desirable material for peripheral nerve repair applications in tissue engineering.

### 3.2. Chitosan-Based Polymer Loading Factors/Cell Repair in PNI

**Figure 4:**
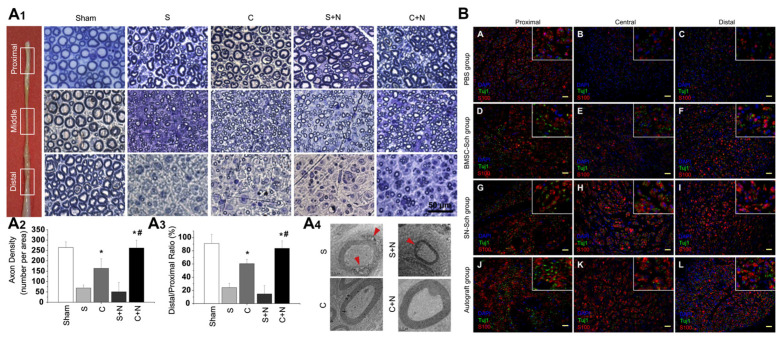
Chitosan-based polymers’ loading factors/cell repair in PNI. (**A**) Morphology of the myelin sheath located in various cross sections of regenerated nerves. (**A1**) Toluidine blue staining demonstrated the disappearance of the myelin sheath in a silicone conduit (S) and the axon re-myelination that occurred in other nerve segments. The representative segments in the proximal, middle, and distal sections of the nerve gap were collected for analysis (indicated by squares). Scale bar, 50 μm. (**A2**) The density of re-myelinated axons in the middle nerve segments (middle portion of conduit) increased in the C and C + N group compared with that of the S group. (**A3**) A similar pattern was also observed in the ratio of re-myelinated axons in the distal segment to those in the proximal segments. (**A4**) Electron microscopy images revealed the degradation of the myelin sheath in the S and S + N groups (arrows). (* *p* < 0.05 compared with the S group; # *p* < 0.05 compared with the C group.) (**B**) Transverse sections of sciatic nerves bridged with PBS (**A**–**C**), BMSC-Sch (**D**–**F**), SN-Sch (**G**–**I**), and autograft (**J**–**L**) at three months post-operation. Copyright reprinted with permission from [29,31].

Chitosan interacts with growth factors and cytokines to enhance the proliferation and differentiation of nerve cells and to regulate the neural regeneration process [14]. As illustrated in Figure 4, chitosan acts as a bioactive material that can sequester and release growth factors and cytokines, providing a localized and sustained delivery system [28,29]. These bioactive molecules play crucial roles in promoting cell proliferation, migration, and differentiation [30]. By interacting with growth factors, chitosan can enhance their bioavailability and facilitate their targeted action on nerve cells, stimulating their proliferation and guiding their differentiation into the specialized cell types necessary for nerve regeneration [31]. Additionally, chitosan’s interaction with cytokines helps regulate the immune response and inflammatory processes associated with nerve injury [32,33]. This modulation of the immune system contributes to creating an optimal environment for neural regeneration. Through its interactions with growth factors and cytokines, chitosan exerts a multifaceted influence on the proliferation, differentiation, and regulation of nerve cells, ultimately enhancing the overall neural regeneration process.

### 3.3. Antimicrobial Properties

Chitosan, a natural polysaccharide, exhibits excellent antibacterial properties. It achieves its antibacterial effects by inhibiting microbial growth and disrupting microbes’ cell wall structure. It demonstrates broad-spectrum antibacterial activity against various pathogenic bacteria, including drug-resistant strains [34,35]. Compared to chemically synthesized antibacterial agents, chitosan has low toxicity and good biocompatibility, with the result that it is widely used in the field of healthcare [36,37]. Additionally, chitosan can form stable films or coatings with long-lasting antibacterial properties, making it suitable for applications in medical devices, food packaging, textiles, and more, effectively preventing microbial growth and transmission [38,39]. In summary, chitosan’s exceptional antibacterial performance establishes it as a crucial antibacterial agent and functional material that positively impacts human health and lifestyle. With the increasing demand for antibacterial solutions, the future of chitosan applications appears even more promising.

### 3.4. Porous Structures for Nutrient and Oxygen Transfer

**Figure 5:**
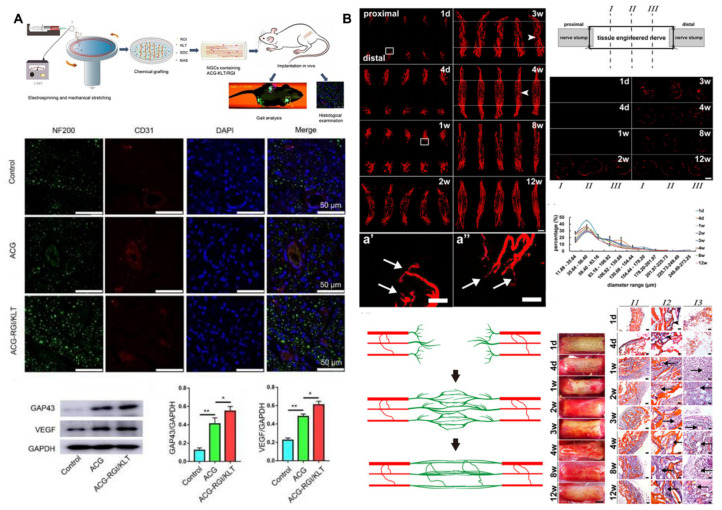
The porous structure of chitosan-based polymers facilitates the transfer of nutrients and oxygen. (**A**) ACG-RGI/KLT promoted angiogenesis and the regeneration of the sciatic nerve. (* *p* < 0.05, ** *p* < 0.01) (**B**) Micro-vessel anastomosis in the middle segment of a tissue-engineered nerve. Red microvessels of the vascular network of a tissue-engineered nerve are displayed. The arrow indicated frontmost branches of sprouting micro vessels. Scale bar, 500 μm in (**a’**) and (**a’’**). Copyright reprinted with permission from [51,53].

Chitosan has a porous structure that facilitates the transport of nutrients and oxygen, promoting the growth of nerve cells and the formation of new blood vessels. The porous nature of chitosan provides a pathway for the efficient diffusion of nutrients and oxygen to the surrounding tissues and cells [40,41]. This superior permeability enables essential nourishment and oxygen to reach the nerve cells, thereby facilitating their growth, differentiation, and regeneration processes [42,43,44]. Additionally, the porous structure of chitosan also supports the formation of new blood vessels [45,46,47,48,49]. The use of achitosan/gelatin thermosensitive hydrogels to promote angiogenesis has recently been proposed by Cheng, Lin, Ling, and Young [50]. As indicated in Figure 5, during the process of nerve regeneration, the formation of new blood vessels is crucial to ensure an adequate blood supply and oxygenation to the nerve cells, promoting their recovery and repair [47,51,52,53,54]. The porous structure of chitosan creates a favorable environment for the transport of nutrients, oxygen, and vascularization, driving the growth and regeneration of nerve cells and facilitating the restoration of neural tissue.

## 4. Chitosan-Based Polymers in Peripheral Nerve Regeneration

The design of materials based on chitosan and its polymers has become an important strategy in promoting the repair of peripheral nerve injuries [55]. These designs can address the specific needs of neural tissue by providing appropriate support and releasing bioactive molecules to facilitate neural regeneration and functional recovery. Another key feature of chitosan and its polymer conduits is their biodegradability. These conduits gradually degrade over time, providing space for the formation of new tissue. This aids in promoting neural regeneration and the repair of the damaged area, while avoiding the adverse effects associated with the long-term presence of the conduits on the surrounding tissues.

### 4.1. Chitosan-Based Polymer Nerve-Repair Conduits

Chitosan and its polymer conduits are typically designed in a tubular structure with controlled inner and outer diameters and lengths [56,57]. The interior of the conduit provides a suitable environment that supports the growth and migration of nerve cells [47]. The porous structure and micro-/nano-scale pores facilitate cell infiltration and provide the necessary support for cell adhesion and directional growth [24].

### 4.2. Chitosan-Loaded Cells

The outer surface of the conduits is often treated to enhance their biocompatibility and cell adhesion [58,59,60,61]. Chitosan and its polymers interact with the surrounding tissues, promoting cell attachment and growth on the conduit’s surface (Table 1) [62]. Chitosan and its polymers have been shown to possess adhesive properties, allowing cells to adhere to their surfaces. This adhesion is crucial during the early stages of nerve regeneration, as it promotes cell migration and proliferation, enabling the formation of a cellular network within the conduit. The ability of chitosan to support cell attachment and growth on the conduit’s surface is attributed to its unique surface chemistry and the presence of functional groups, such as amino and hydroxyl groups, which facilitate cell-substrate interactions. This aids in establishing stable contact with the surrounding neural tissue, facilitating neural regeneration.

### 4.3. Chitosan Slow-Release Bioactive Molecules

Conduits made of chitosan and its polymers can promote cell proliferation and differentiation by adsorbing and releasing bioactive molecules (Table 2) [79,80,81]. These bioactive molecules may include nerve growth factors, cell adhesion molecules, and drugs, which play important regulatory roles in neural regeneration processes [82,83,84]. According to the present study, the nerve growth factor (NGF), the neurotrophic factor-3 (NT-3), the neurotrophic factor-4 (NT-4), the brain-derived nerve growth factor (BDNF), and the vascular nerve growth factor (VEGF) are among the molecules that can promote peripheral nerve regeneration. The design of the conduits allows for the incorporation or coating of these molecules on the conduit’s surface, providing continuous bioactive signaling to promote cell growth and functional recovery.

## 5. Techniques of Tissue Engineering for PNI Repair

Chitosan-based hydrogels provide an ideal environment for nerve regeneration, as they exhibit excellent biocompatibility and biodegradability. Three-dimensional printing enables the fabrication of customized biological scaffolds to support nerve regeneration. Electrospun chitosan fiber scaffolds, produced using electrospinning technology, promote the regeneration of nerve cells. The application potential and versatility of chitosan offer new opportunities in the field of nerve regeneration.

### 5.1. Hydrogel for PNI Repair

**Figure 6:**
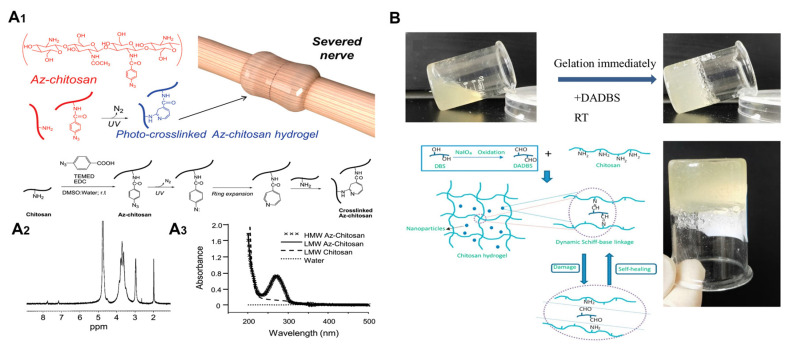
Chitosan-based hydrogels for peripheral nerve repair. (**A**) Schematic of the conjugation of chitosan with ABA and the photo-cross-linking of Az-chitosan chains. (**A1**). The 1H NMR spectra (**A2**) were identical for LMW and HMW Az-chitosan. Similarly, the UV/vis absorbance spectra (**A3**) of LMW and HMW Az-chitosan in water (1 mg/mL, pH 5) overlapped. (**B**) Photographs of a medium molecular weight chitosan hydrogel formed immediately after mixing 4% chitosan solution and DADBS at 25 °C (the molar ratio of aldehyde to the amino group was 1.4). copyright reprinted with permission from [117,120].

Chitosan-based shell hydrogels have emerged as a promising and innovative approach for the treatment of peripheral nerve injuries. As shown in Figure 6, these hydrogels offer excellent biocompatibility and biodegradability, creating an optimal environment for nerve cell growth and regeneration [83,116,117]. They provide a supportive three-dimensional network structure and regulate the release of bioactive molecules, such as nerve growth factors, to promote nerve regeneration and improve the microenvironment at the injury site [118,119]. The porous structure of the hydrogels facilitates cell invasion and guided growth, enabling nerve cells to reconnect within the injury site [2,120,121]. The adhesive properties of the hydrogels promote cell attachment and growth, accelerating the process of nerve regeneration [122,123]. Moreover, the biodegradability of the hydrogels eliminates the need for surgical removal and allows for the formation of new tissue. In addition, chitosan-based hydrogels exhibit excellent antimicrobial properties that can reduce the potential for implant infection [124]. While further research is needed to fully understand their mechanisms and optimize their therapeutic potential, chitosan-based shell hydrogels hold show great promise for the enhancement of nerve regeneration and functional recovery. Their unique properties make them a valuable tool in the treatment of peripheral nerve injuries, offering new possibilities for improving clinical outcomes and revolutionizing nerve repair strategies.

### 5.2. Three-Dimensional Printing Technology Applied to PNI Repair

**Figure 7:**
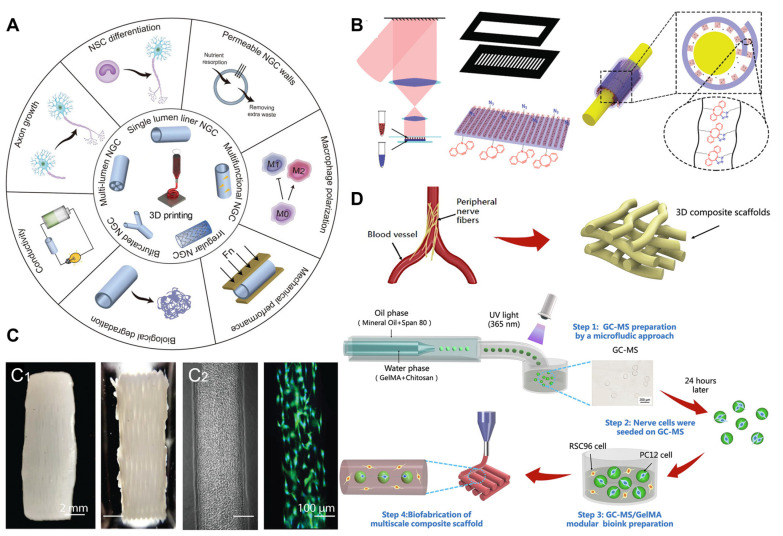
Three-dimensional-printed chitosan-based biopolymers for peripheral nerve repair. (**A**) Three-dimensional NGCs are printed for nerve regeneration. Various types of NGCs with different topological structures and physical properties are manufactured via three-dimensional printing for the effective repair of nerve defects within complex anatomical structures. (**B**) A three-dimensional-printed self-adhesive drug-loaded bandage surrounding a nerve and releasing drugs. (**C**) three-dimensional-printed hydrogel for implantation. (**C1**) Fibrin/HA scaffold containing encapsulated Schwann cells via a one-pot printing process. (**C2**) Phase contrast and fluorescence images of printed Schwann cells in a 200 μm width channel. (**D**) Confocal microscopy of the cells before and after the application of mechanical or electrical cues. Copyright reprinted with permission from [127,129,130,131].

The three-dimensional printing of chitosan-based materials is an emerging technology for the treatment of peripheral nerve injuries. By utilizing three-dimensional printing techniques, customized chitosan conduits, scaffolds, and implants with intricate structures and precise dimensions can be fabricated, providing individualized solutions for nerve regeneration [125,126]. These chitosan-based conduits possess unique structures and a porous architecture, which offer excellent support and guidance for neural cells, promoting their regeneration and connection within the injury site [127,128]. During the three-dimensional printing process, chitosan-based materials can be combined with other biocompatible materials to further enhance their biocompatibility and mechanical properties, as shown in Figure 7 [129]. By manipulating the composition and printing parameters, the pore structure, mechanical characteristics, and release behavior of the conduits can be controlled to meet the specific requirements of the neural tissue. This personalized treatment approach improves the success rate of nerve regeneration and patient rehabilitation outcomes [130]. However, the three-dimensional printing of chitosan-based materials still faces challenges when used in the treatment of peripheral nerve injuries; these challenges include the biodegradability of the materials, the long-term stability of the conduits, and interactions with the surrounding tissues. Therefore, further research and clinical practice are needed to validate their safety and efficacy. Overall, the three-dimensional printing of chitosan-based materials provides a cutting-edge solution for the treatment of peripheral nerve injuries, and the personalized design and bioactivity of these materials make them a powerful tool for promoting neural regeneration and recovery. With continued advancements in research and technology, it is believed that the three-dimensional printing of chitosan-based materials will facilitate further breakthroughs and innovations in the field of neuroscience.

### 5.3. Electrostatic Spinning for PNI Repair

**Figure 8:**
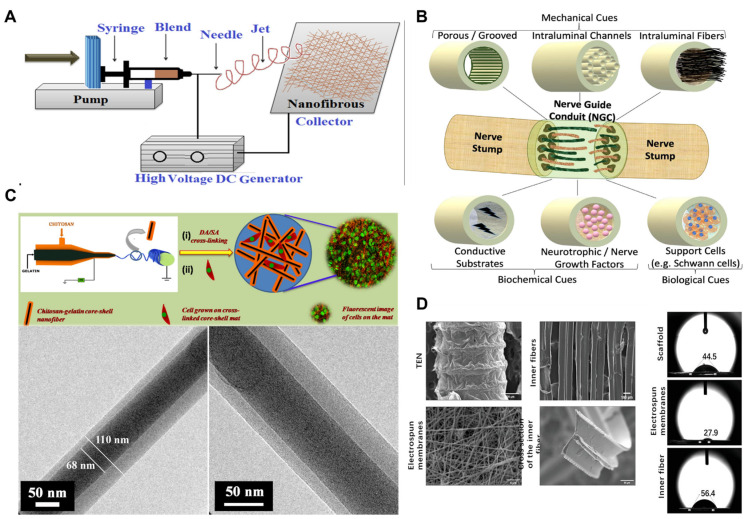
Electrospun chitosan-based biopolymers for peripheral nerve repair. (**A**) Schematic diagram of the electrospinning process for producing nanofibers. (**B**) Nerve guide conduits (NGCs) with mechanical, biochemical, and biological cues. (**C**) TEM micrographs of gelatin/chitosan core–shell nanofibers. (**D**) Structure of the phthalated cashew gum (PCG) and chitosan (CH) polymers. Copyright reprinted with permission from [132,135,136,137].

The electrospinning of chitosan-based materials has emerged as a promising approach for the treatment of peripheral nerve injuries. This advanced fabrication technique enables the production of fine and intricately structured fiber scaffolds that provide an optimal environment for nerve regeneration [132]. As shown in Figure 8, electrospun chitosan-based materials possess a high surface area-to-volume ratio and a porous structure, facilitating cell attachment, growth, and differentiation, while exhibiting excellent biocompatibility and biodegradability [133]. By controlling the electrospinning parameters and chitosan concentration, the diameter of the fiber scaffolds, fiber spacing, and pore size can be tailored to meet the specific requirements of peripheral nerve tissues [134]. Additionally, chitosan-based materials can be further enhanced by drug loading or the incorporation of bioactive molecules such as growth factors and cell adhesion molecules to promote nerve regeneration and repair. These bioactive molecules can be incorporated into the fiber scaffolds during the electrospinning process and released in a sustained manner, providing appropriate signaling cues during the treatment. The application of electrospun chitosan-based materials shows great promise for the treatment of peripheral nerve injuries. However, challenges such as the mechanical stability and consistency of the fiber scaffolds, as well as the material’s uniformity and scalability, need to be addressed to advance the widespread clinical utilization of electrospun chitosan-based materials. Overall, the electrospinning of chitosan-based materials shows significant potential to enhance the treatment and regeneration of peripheral nerve injuries, contributing to important breakthroughs and innovations in the field of neuroscience.

## 6. Conclusions and Future Prospects

Chitosan-based polymers are a promising material that can promote the repair and regeneration of peripheral nerves. They have been extensively studied and applied in the field of nerve regeneration. Chitosan-based polymers possess good biocompatibility and biodegradability, allowing them to provide support and protection for damaged nerve tissues. Chitosan-based hydrogels can form soft gels with high water content. They exhibit excellent biocompatibility and mechanical properties, mimicking the characteristics of biological tissues. Moreover, three-dimensional printing technology can be used to construct nerve regeneration scaffolds using chitosan-based polymer materials. Through three-dimensional printing, the shape and structure of the materials can be precisely controlled to manufacture customized biomaterial scaffolds that support and guide nerve regeneration according to individual needs. Electrospinning can be utilized to prepare chitosan fiber scaffolds with a high surface area and a nanoscale structure. These fiber scaffolds can serve as templates for nerve regeneration, providing surfaces for cell adhesion and growth to facilitate the regeneration and connection of nerve cells. In conclusion, chitosan-based polymers are multifunctional materials that can be applied in the field of nerve regeneration by using techniques such as hydrogels, three-dimensional printing, and electrospinning. Their unique properties and versatility make them a promising material for promoting the repair and functional recovery of peripheral nerves. Future research and applications will further drive the development of chitosan-based polymers in the field of nerve regeneration.

## Figures and Tables

**Table 1:** Cell types that can be loaded with chitosan-based biopolymers for peripheral nerve injury repair.

Cell Types	Origins	Characteristics	Applications	Reference
Adult Neural Stem Cells	Brain and bone marrow	Self-renew and differentiate into various types of neural cells	Promote neural regeneration and repair	[63]
Human Embryonic Stem Cells	Early-stage human embryos	Broad differentiation potential, capable of generating various cell types	Repair peripheral nerves	[64,65]
Induced Pluripotent Stem Cells	Reprogrammed from adult body cells	Induced to differentiate into neural cells	Treat neural injuries and diseases	[66,67,68,69]
Adipose-Derived Stem Cells	Human adipose tissue	Relatively easy to obtain and expand	Promote neural regeneration	[70,71]
Peripheral Neural Stem Cells	Peripheral nervous system	Differentiate into various types of neural cells	Transplantation and facilitating neural regeneration and repair	[72,73,74]
Astrocytes	Neural progenitor cells	Provide support and nourishment for neuronal survival	Provide structural support, regulate the chemical environment	[75,76]
Schwann Cells	Neural crest	Generate myelin sheaths and promote neuronal regeneration and repair	Wrap around peripheral nerve fibers and form myelin sheaths	[77,78]

**Table 2:** Types of factors that can be loaded by chitosan-based biopolymers for peripheral nerve injury repair.

Factor Types	Including	Origins	Characteristics	Reference
Neurotrophic Factors	Nerve growth factor (NGF), brain-derived neurotrophic factor (BDNF), neurotrophin-3 (NT-3), etc.	Neurons, astrocytes, and immune cells.	Facilitate neurite outgrowth, enhance neuronal cell survival and function	[85,86,87]
Inflammatory Factors	Tumor necrosis factor-alpha (TNF-α), interleukin-1 beta (IL-1β)	Macrophages and lymphocytes.	Promote the occurrence and regulation of inflammatory responses, influencing neural repair	[88,89,90]
Cell Adhesion Molecules	Neural cell adhesion molecule (NCAM), integrins, and selectins	Nerve cells	Regulating neuronal migration, positioning, and connectivity	[91,92,93]
Matrix Metalloproteinases	Enzymes	Macrophages, astrocytes, and endothelial cells	Cell migration and neurite formation	[94,95]
Angiogenic Factors	Vascular endothelial growth factor (VEGF), basic fibroblast growth factor (bFGF)	Endothelial cells, astrocytes, and inflammatory cells	Promote the growth of blood vessels, increasing the supply of oxygen and nutrients	[96,97,98,99]
Immunomodulatory Factors	Interleukin-10 (IL-10), transforming growth factor-beta (TGF-β)	Immune cells, astrocytes	Regulate immune responses, inhibit excessive immune reactions and inflammation	[100,101,102,103,104]
Fibrotic Factors	Collagen proteins, fibronectin,	Fibroblasts and inflammatory cells	Hinder scar formation and impede neural regeneration	[105,106,107]
Cytokines	Immune cells, astrocytes, and neurons	Interleukins, interferons,	Regulation of immune and inflammatory responses	[108,109,110,111]
Exosomes	Proteins, nucleic acids, and lipids	Neural cells, astrocytes, and immune cells	Intercellular communication, transferring functional molecules and signals between cells	[66,112,113,114,115]

## Data Availability

The data presented in this study are available on request from the corresponding author.
